# Microglial morphology aligns with vigilance stage‐specific neuronal oscillations in a brain region‐dependent manner

**DOI:** 10.1002/glia.24617

**Published:** 2024-09-20

**Authors:** Sarah Steffens, Hilla Mäkinen, Tarja Stenberg, Henna‐Kaisa Wigren

**Affiliations:** ^1^ SLEEPWELL Research Program I Faculty of Medicine University of Helsinki Finland; ^2^ Molecular and Integrative Biosciences Research Programme I Faculty of Biological and Environmental Sciences University of Helsinki Finland

**Keywords:** EEG, microglia morphology, oscillations, vigilance stages

## Abstract

Microglia, the resident immune cells in the brain, dynamically adapt their morphology based on their functional state. This study explored the relationship between microglial morphology and sleep–wake cycles in mice. Using Iba1 immunostaining to identify microglia, we quantified morphological changes in microglia at different timepoints in multiple brain regions (cortex, hippocampus, basal forebrain, hindbrain, and cerebellum) in B6 male mice using semi‐automated 3D structural analysis. Simultaneously, in a separate group, we monitored wake and sleep stage‐specific brain activity using EEG/EMG recordings. During natural sleep–wake cycles, we observed increased microglial complexity (enlarged volume, territorial coverage, and ramification) during wakefulness, characterized by high‐frequency theta (8–12 Hz) and gamma activity (30–80 Hz). Conversely, during NREM sleep, which is dominated by delta activity (0.5–4 Hz), microglia displayed reduced complexity. Notably, this pattern was absent in brain regions lacking direct functional connections to areas generating vigilance stage‐dependent thalamocortical oscillations. We then extended wakefulness to decouple circadian influence from sleep–wake‐specific neuronal activity. This procedure attenuated the decrease in microglial complexity observed during natural sleep, suggesting a crucial role for neuronal activity. Subsequent recovery sleep restored microglial features, independent of the time of day (zeitgeber time). These findings reveal a dynamic interplay between vigilance stage‐specific thalamocortical activity and microglial morphology across various brain regions. This suggests a potential role for microglia in sleep regulation and warrants further investigation to understand the underlying mechanisms.

## Main Points

- Microglia complexity ↑ during wakefulness (theta/gamma activity) & ↓ during NREM (delta activity).
- Effect is lost in brain areas without connection to vigilant stage‐specific activity.
- Extending wakefulness attenuated ↓ in microglial complexity.

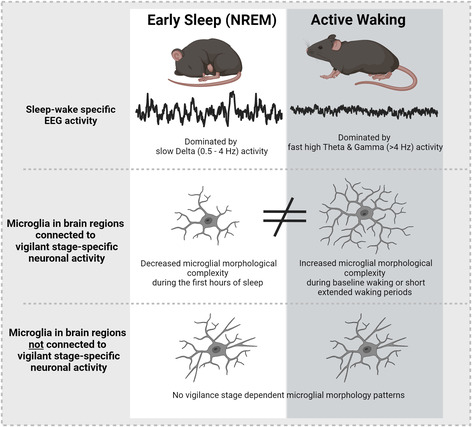

**Abbreviations**
3Dmorph: software for 3D analysis of microscopy images
3EW and 9EW: extended wakefulness for 3 h and 9 h
BF: basal forebrain
CC: cerebellar cortex
CNS: central nervous system
CSF1R: colony‐stimulating factor 1 receptor
Ctr: control
DCN: deep cerebellar nucleus
EDTA: ethylenediaminetetraacetic acid (anticoagulant)
EEG: electroencephalogram
EMG: electromyography
EWrec: recovery sleep after extended wakefulness
FFT: fast Fourier transformation
GEEs: generalized estimating equations
HC: dorsal hippocampus
MVN: medial vestibular nucleus
NGS: normal goat serum (used in immunohistochemistry)
NREM: non‐rapid eye movement sleep
PBS: phosphate buffered saline
PFA: paraformaldehyde
REM: rapid eye movement sleep
SC: somatosensory cortex
SEM: standard error of the mean
SF: sleep fragmentation
SWA: slow wave activity
TBS‐T: Tris‐buffered saline with Tween
ZT: zeitgeber time

## 1 INTRODUCTION

The stages of sleep and wakefulness are characterized by specific neuronal oscillations in the brain. The oscillations are measured using cortical electroencephalogram (EEG) and subdivided into different thalamocortical oscillation frequencies (as reviewed in Adamantidis et al., 2019). Waking is dominated by fast theta (8–12 Hz) and gamma activities (30–80 Hz) (Buzsáki & Moser, 2013; Huber et al., 2007; Jones, 2020), while rapid‐eye‐movement sleep (REM) is dominated by theta activity (5–12 Hz), and non‐REM sleep (NREM) is characterized by delta or slow wave activity (SWA) (0.5–4 Hz). The SWA arises from synchronized thalamocortical network activity, propagating to other brain regions (Adamantidis et al., 2019). However, some cerebellar/hindbrain areas lack strong functional connections to the reticular thalamic areas that produce vigilance stage‐specific oscillations (Barmack, 2003; Cullen & Roy, 2004).

Sleep, according to the 2‐process model, is regulated by two key factors: the circadian and homeostatic processes (Achermann et al., 1993; Borbély, 1982; Borbély & Tobler, 2023). Sleep pressure, measured by SWA, accumulates with wakefulness and triggers sleep initiation. Increases in wake intensity, measured by theta and gamma activities (theta‐dominated wakefulness, TDW) (Vassalli & Franken, 2017), increases sleep‐pressure accumulation (Hubbard et al., 2020; Huber et al., 2007; Vyazovskiy et al., 2004). Extended wakefulness further increases sleep pressure, which upon falling asleep is followed by excessive SWA (recovery sleep). However, some sleep restriction protocols, like fragmentation or those ending during circadian activity periods, do not induce recovery sleep (Franken & Dijk, 2023; Ramesh et al., 2012).

The other sleep regulator, the circadian process, driven by the suprachiasmatic nucleus, controls arousal (Hastings et al., 2018; Mohawk et al., 2012). These two processes work together to regulate sleep timing and duration. However, the processes can be separated, for example, by using the constant routine protocol (Dijk et al., 1992). Extended wakefulness (3–9 h) builds excessive sleep pressure, but affects the circadian rhythm only minimally (Franken & Dijk, 2023). Sleep fragmentation (1–2 weeks) does not induce a SWA increase upon return to a normal sleep schedule (Ramesh et al., 2009).

Emerging evidence implicates glia in regulating brain functions (Artiushin & Sehgal, 2020; Bellesi et al., 2017; 2020). While astrocytes are known to play a role in the development of sleep pressure and sleep‐related neuronal synchrony (Fellin et al., 2012), the involvement of microglia remains less understood.

Microglia are the resident immune cells in the central nervous system and make up 5%–12% of the total mouse brain cells (Lawson et al., 1992; Li & Barres, 2018). Pathological challenges induce a microglial state change, including a change of their morphology, gene expression profile, proliferation rate, migration to sites of damage, increase of phagocytic activity, and release of inflammatory mediators, for example, cytokines (Loane & Kumar, 2016; Matsudaira & Prinz, 2022; Paolicelli et al., 2022; Perry et al., 2003; Xu et al., 2016), accompanied by a morphological change of decreased ramification and increased cell soma size (Augusto‐Oliveira et al., 2022; Savage et al., 2019).

Microglial morphology varies across the diurnal rhythm and sleep–wake cycle (Garofalo et al., 2020; Hristovska et al., 2022; Nakanishi et al., 2021; Steffens et al., 2022). In male mice, microglia expressed less ramification during the light (when mice are mainly behaviorally inactive) than during the dark (behaviorally active) phase in the somatosensory cortex (SC) (Hayashi, Koyanagi, Kusunose, Okada, et al., 2013), hippocampus, and basal forebrain (BF) (Steffens et al., 2022). Additionally, increased ramification during wakefulness has previously been reported (Hristovska et al., 2016, 2022). The question regarding whether these changes are more sleep–wake cycle or circadian time‐related has not been systematically studied.

Our previous work revealed that microglial morphology varies throughout the day (Steffens et al., 2022). Here we investigate whether, and to what extent, these changes reflect responses to neuronal activity patterns associated with vigilance stages.

Our hypothesis is that microglial activity, as reflected in its morphology, is regulated by the circadian and homeostatic factors, where the homeostatic factor is represented by neuronal activity.

To investigate this, we used different sleep modulation protocols that are known to induce different homeostatic responses, enabling inspection of the circadian and homeostatic factors separately.

Further, we focused on brain regions with both weak and strong connections to vigilance‐related thalamocortical networks. We compared the morphology in the cortex, hippocampus, and BF (areas with strong thalamocortical connections) to the cerebellar cortex (CC), medial (fastigial) part of the deep cerebellar nucleus (DCN) and medial vestibular nucleus (MVN) (weak or nonexistent connections) across different vigilance stages.

## 2 METHODS

### 2.1 Animals and light conditions

We used inbred wild type C57BL/6JRccHsd (B6) male mice (*N* = 64) purchased from Envigo (Limburg, Netherlands) and maintained at the University of Helsinki Laboratory Animal Center. We started the experimental procedures at the age of 10 ± 2 weeks and body weight of 28 ± 3 g. All animals were group‐housed, except for the animals implanted with the electroencephalographic/electromyographic (EEG/EMG) transmitters, since postsurgery recovery was not possible in a group. The room temperature was controlled (22 ± 2°C) and light–dark cycle set to 12:12 lights‐on (light intensity 100 lx) at zeitgeber time 0 (ZT0) and lights‐off at ZT12. Mice had access to food and water *ad libitum*. To avoid any selection bias, the animals were randomly allocated to the treatment groups and their respective controls, and the resulting groups were adjusted for similar body weight distribution at the start of the study.

All animal procedures were approved by the Regional state Administration Agency for Southern Finland (ESAVI/9056/2020) and conducted in accordance with the Finnish Act on the Protection of Animals used for Science or Educational Purposes and in accordance with directive 2010/63/EU of the European Parliament and of the Council. All efforts were made to minimize the number of animals used and their suffering.

### 2.2 Experimental groups and sleep manipulations

**FIGURE 1:**
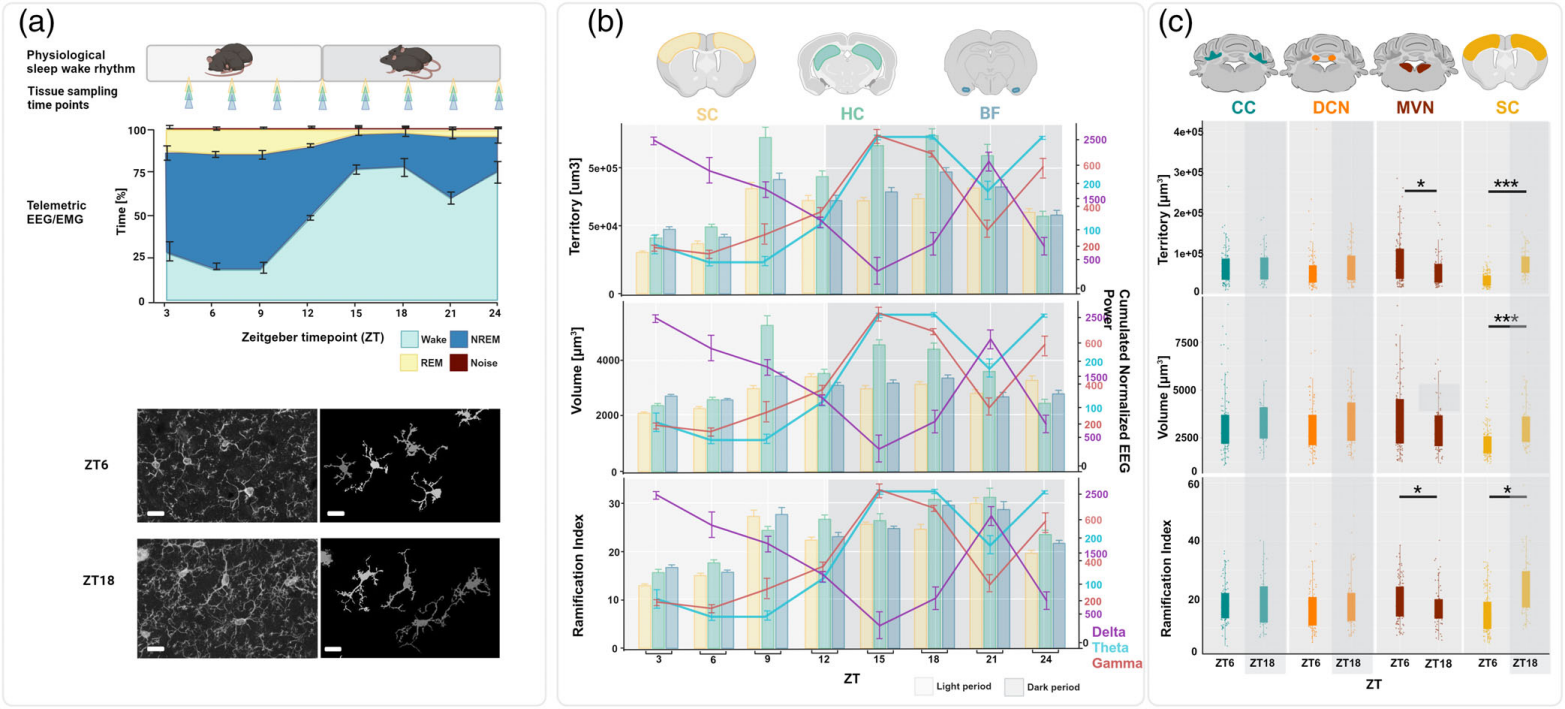
Baseline measurements. (a) We imaged microglia in brain samples collected every 3 h across a 24‐h period with a species‐specific distribution of vigilance stages (*N*_animals_ = 6: wake [light blue], non‐rapid eye movement sleep [NREM, dark blue], and rapid eye movement sleep [REM, yellow]). Representative maximum‐projections of the confocal z‐stacks and 2D reconstructions of microglial cells at the timepoint of minimum (ZT6) and maximum (ZT18) complexity in the somatosensory cortex (SC). Scale bars = 10 μm. (b) The pattern of microglial morphological changes in SC (yellow), hippocampus (HC, green), and basal forebrain (BF blue) as compared with the diurnal pattern of the vigilance stage‐specific EEG power, including NREM delta (0.5–4 Hz, purple), waking high frequency theta (7–12 Hz, light blue), and waking gamma (30–80 Hz, red). (c) Hindbrain (DCN and MVN) and cerebellar (CC) microglia did not display similar differences in their morphology between light (ZT6) and dark (ZT18) periods as detected in the cortex (SC). Samples from the CC (cyan), DCN (orange), MVN (brown), and SC collected from ZT6 (light period) were compared with those from ZT18 (dark period). (ZT6, *N*_animals_: CC = 4, DCN = 4, MVN = 4, SC = 5; N cells: CC = 47, DCN = 70, MVN = 70, SC = 70; ZT18, *N*_animals_: CC = 6, DCN = 6, MVN = 6, SC = 9; *N*_cells_: CC = 102, DCN = 84, MVN = 101, SC = 142). Statistical significance between the timepoints is indicated with asterisks (*p ≤ 0.05; **p ≤ 0.01; ***p ≤ 0.005). EEG, electroencephalogram; EMG, electromyography.

We used one baseline group (Figure 1a) and four experimental procedures with their corresponding control groups to manipulate sleep, recovery sleep and its timing.

**FIGURE 2:**
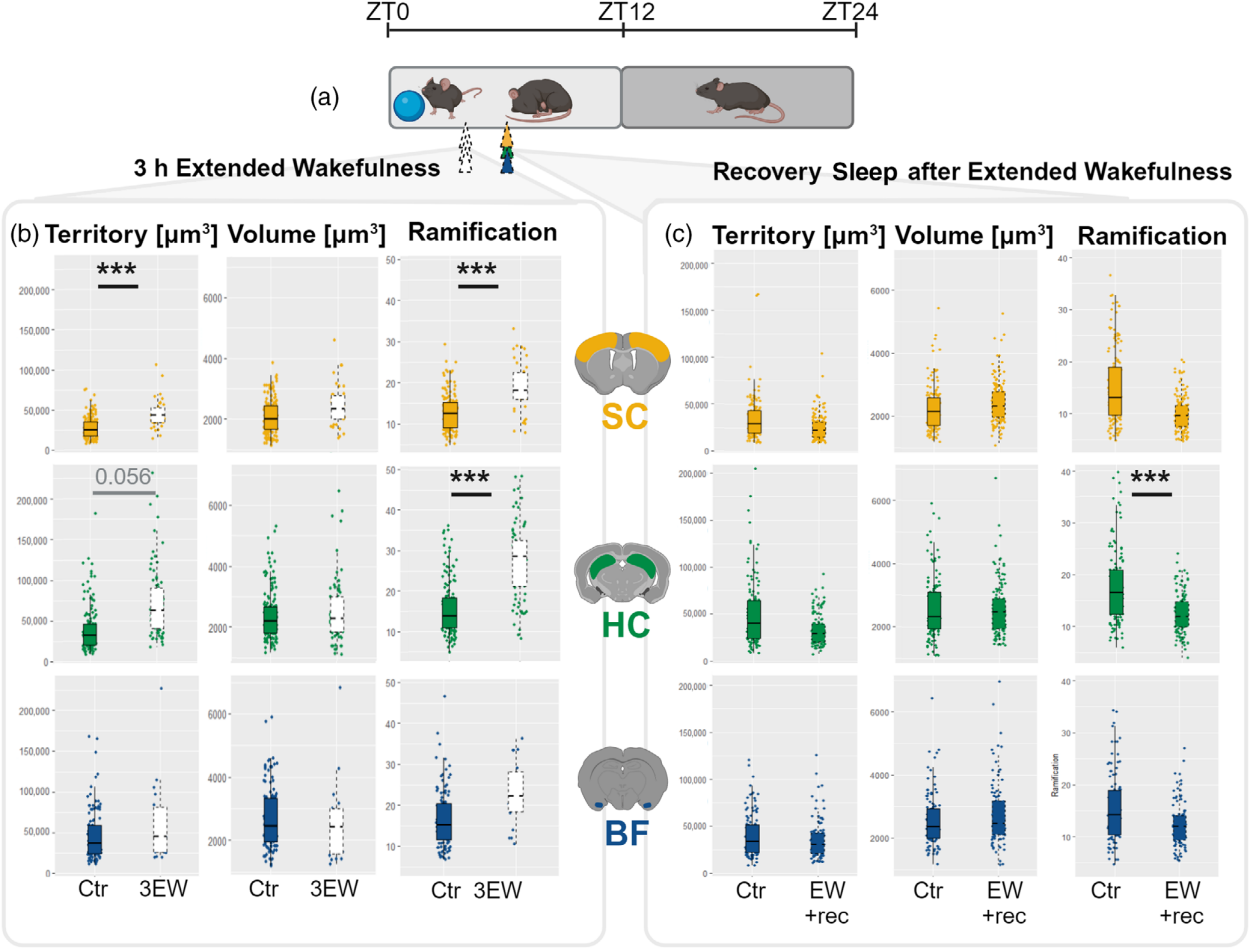
Effects of extended wakefulness and the following recovery sleep on microglial morphology in the beginning of the light period. (a) Samples from the SC (yellow), HC (green), and BF (blue) were collected after extended wakefulness (3EW) at ZT3, and after 2 h of recovery sleep (EW‐rec) at ZT6 and compared with undisturbed controls (Ctr) collected at corresponding timepoints. (b) After 3 h of extended wakefulness at ZT3, microglial territory and ramification complexity increased in SC and HC (3EW, *N*_animals_: SC = 4, HC = 6, BF = 4; *N*_cells_: SC = 38, HC = 77, BF = 19; 3EW‐Ctr, *N*_animals_: SC = 8, HC = 7, BF = 8; *N*_cells_: SC = 146, HC = 146, BF = 114). (c) After 2 h of recovery sleep at ZT6, microglial territory and ramification complexity returned to control values or in HC even lower as compared with their Ctrs (EW‐rec, *N*_animals_: SC = 5, HC = 5, BF = 5; *N*_cells_: SC = 147, HC = 123, BF = 100; EW‐rec‐Ctr, *N*_animals_: SC = 9, HC = 10, BF = 11; *N*_cells_: SC = 142, HC = 129, BF = 111). Statistical significance between experimental groups and their respective controls is indicated with asterisks (*p ≤ 0.05; **p ≤ 0.01; ***p ≤ 0.005). EEG, electroencephalogram; EMG, electromyography.

**FIGURE 3:**
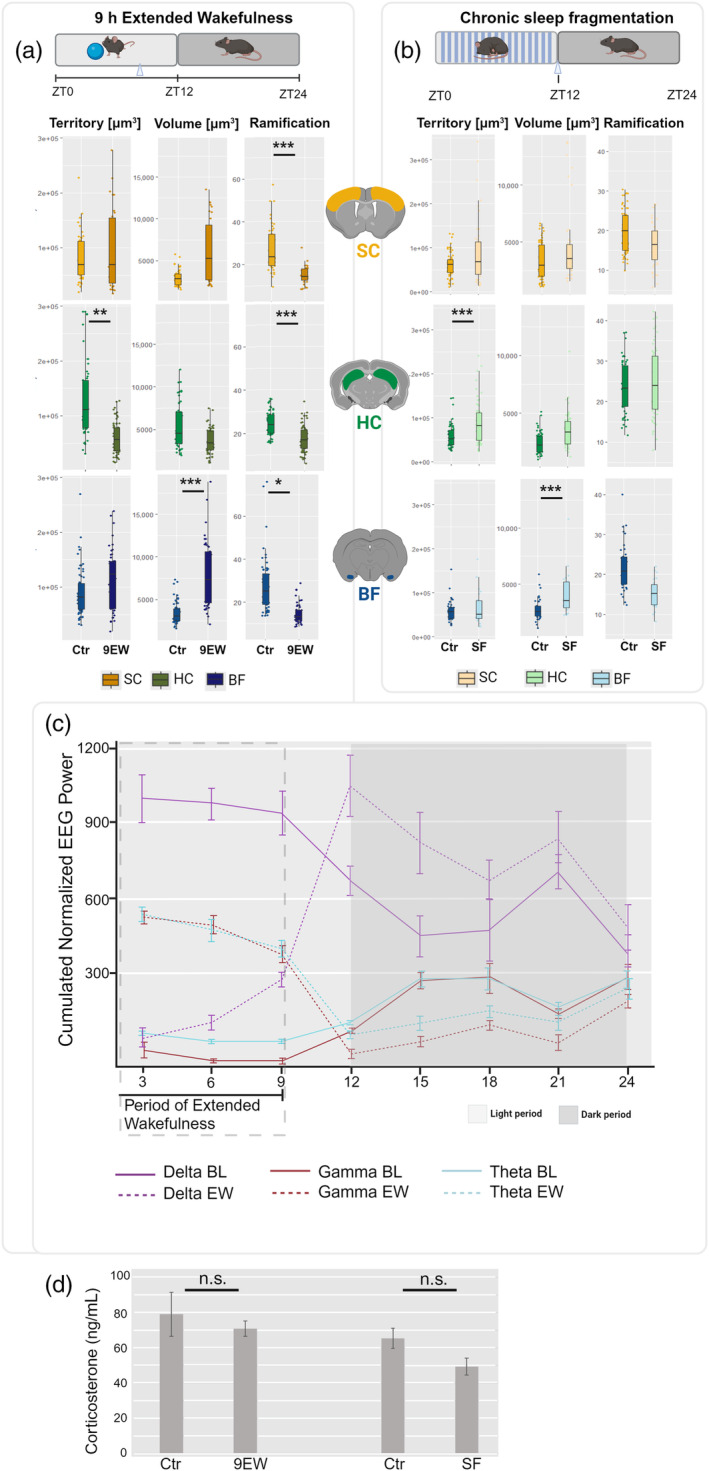
Microglial morphology after 9 h of SD and after 14 days SF. We collected samples from the SC (yellow), HC (green) and BF (blue) after 9 h of extended wakefulness (9EW) at ZT9 (a) and after 14 days of chronic sleep fragmentation (SF) at ZT0 (b) and compared them with undisturbed controls (Ctr) collected at corresponding timepoints. (a) 9EW decreased microglial ramification complexity in the SC, HC, and BF, decreased the territory in the HC and increased volume in the BF (9EW, *N*_animals_: SC = 6, HC = 8, BF = 7; *N*_cells_: SC = 31, HC = 65, BF = 45; 9EW‐Ctr, *N*_animals_: SC = 4, HC = 5, BF = 7; *N*_cells_: SC = 46, HC = 53, BF = 60). (b) SF had only minor effects on microglial morphology, including an increased territory in the HC and an increased volume in the BF (SF, *N*_animals_: SC = 8, HC = 11, BF = 4; *N*_cells_: SC = 45, HC = 59, BF = 22; SF‐Ctr, *N*_animals_: SC = 6, HC = 7, BF = 8; *N*_cells_: SC = 63, HC = 54, BF = 37). (c) The vigilance stage‐specific EEG power during and after extended wakefulness as compared with baseline, including NREM delta (0.5–4 Hz, purple), waking high frequency theta (7–12 Hz, light blue), and waking gamma (30–80 Hz, red) (*N*_animals_ = 6). (d) Blood‐sampled corticosteroid levels did not increase by 9EW or after SF (*N*_animals_ = 6). Statistical significance between experimental groups and their respective controls is indicated with asterisks (*p ≤ 0.05; **p ≤ 0.01; ***p ≤ 0.005). EEG, electroencephalogram; EMG, electromyography.

1. 3 h extended wakefulness (3EW) (Figure 2a): Mice were kept awake for 3 h by the gentle handing method (Franken et al., 1991) starting at lights on at ZT0 until ZT3. Their control group (3EW‐Ctr) was left undisturbed. Both groups were sacrificed at ZT3.
2. 2 h of recovery sleep after extended wakefulness (EWrec) (Figure 2a): Another group of mice was allowed 2 h of undisturbed recovery sleep after 4 h of extended wakefulness. Their control group (EWrec‐Ctr) was left undisturbed the entire time, ZT0–ZT6. Both groups were sacrificed at ZT6.
3. 9 h extended wakefulness (9EW) (Figure 3a): We used the gentle handling method to keep the animals awake starting at lights on at ZT0. The control group (9EW‐Ctr) was left undisturbed. Both groups were sacrificed at ZT9.
4. Chronic sleep fragmentation (SF) (Figure 3b): For 2 weeks, we performed this experimental condition with the help of the SF chamber (Model 80391, Lafayette Instruments, Lafayette, Indiana, USA) (Hakim et al., 2015; Kaushal et al., 2012) deploying an automated sweeper arm, set to briefly interrupt the animals' sleep every 2 min during the lights‐on period (ZT0–ZT12) to induce SF. For habituation, the SF mice were group‐housed in the SF chamber for 5 days prior to the experiment, with three additional days of sweeper arm activity at a 20‐min interval during the dark phase. Control group (SF‐Ctr) animals were group‐housed under the same conditions, but with the sweeper arm inactive or in regular, individually ventilated cages (IVC). Both groups (SF and SF‐Ctr) were perfused after the 2 weeks at the end of the last dark period at ZT0.

### 2.3 Sample collection

The cages of control and treatment groups were placed in an alternating fashion in the animal rooms to guarantee similar lighting and temperature conditions and selected in a random order from the treatment and control groups to control for sampling bias. Perfusions were performed as described in (Steffens et al., 2022): the mice were injected with a lethal dose of pentobarbital (120 mg/kg body weight, Mebunat Vet, Orion Pharma, Espoo, Finland), transcardially perfused with 4% paraformaldehyde (PFA, 37% PFA diluted in phosphate‐buffered saline [PBS, 0.1 M, pH 7.4]) and the brains postfixed in 4% PFA for 24 h, followed by 48 h incubation in 30% sucrose, and stored at −80°C.

### 2.4 Microglial morphology: Immunohistochemistry, image acquisition, and analysis

Coronal sections (35 μm) from the SC, dorsal hippocampus (HC), BF, CC, medial (fastigial) part of the DCN, and MVN (distance from the Bregma; SC: 0.34 mm; HC: 2.30 mm; BF: 0.14 mm, CC, DCN and MVN: −6 mm) were sliced using a Leica CM3050 S cryostat and collected into Tris‐buffered saline with Tween (TBS‐T; pH 7.2; 0.01 M with 0.05% Tween) and immunohistochemically stained for microglial cells as in (Steffens et al., 2022): free‐floating sections were blocked against nonspecific binding with 10% goat serum (NGS, Jackson ImmunoResearch, West Grove, Pennsylvania, USA) in TBS‐T for 90 min, incubated with the primary antibody (polyclonal rabbit anti‐IBA1 IgG antibody 1:2000, 234 003, Synaptic Systems, Göttingen, Germany) at 4°C for 18 h and subsequently with the secondary antibody (AlexaFluor 568 goat anti‐rabbit 1:500; SySy, Göttingen, Germany) for 2 h at room temperature. The brain slices were then mounted on microscope slides (Menzel, 76 × 26 mm) and coverslipped with ImmuMount (both Thermo Fisher Scientific, Vantaa, Finland) and air‐dried.

Five images per brain area per animal were acquired with a SP8 confocal microscope (Leica Microsystems, Wetzlar, Germany) using 63× magnification and the z‐stack function (step size 0.2 μm; 55 to 80 images per stack). Microglial cells were traced with the Matlab‐based 3Dmorph (York et al., 2018) as in (Steffens et al., 2022).

Microglial morphology was assessed as defined by York et al. (2018), a Matlab‐based program that semiautomatically processes individual microglial morphology from overlapping 3D clusters, measuring territory (volume of a 3D polygon around the cell's external points), volume (number of voxels multiplied by the scale to convert into real‐world units), and ramification complexity (ratio of the cell's territory to its volume), three features that have previously been shown to significantly vary at different times of day and with different vigilance stages (wakefulness, NREM, REM) (Steffens et al., 2022).

Some samples were excluded from the final analysis due to insufficient staining quality or poor image resolution that compromised the ability to accurately assess microglial features. The chosen quantification method, 3DMorph, excels in its unbiased, automated, and detailed analysis of microglial morphology. However, it is also sensitive to minor variations in image quality. Consequently, the following number of animals was excluded from each group:

- 3EW versus 3EW‐Ctr: SC: 2 excluded, HC: 0 excluded, BF: 2 excluded (originally 6;6;6 + 8;8;8, final: 4;6;4 + 8;7;8)
- 3EWrec versus 3EWrec‐Ctr: SC: 1 excluded, HC: 1 excluded, BF: 1 excluded (originally 6;6;6 + 11;11;11, final: 5;5;5 + 9;10;11)
- 9EW versus 9EW‐Ctr: SC: 2 excluded, HC: 0 excluded, BF: 1 excluded (originally 8;8;8 + 8;8;8, final: 6;8;7 + 4;5;7)
- SF versus SF‐Ctr: SC: 3 excluded, HC: 4 excluded, BF: 4 excluded (originally 11;11;11 + 8;8;8, final: 8;11;4 + 6;7;8)

Image processing and cell tracing were performed blinded to the experimental condition.

### 2.5 Plasma corticosteroid measurements

For collection of plasma corticosteroid samples after 9 h of EW at ZT9 and after 2 weeks of SF at ZT0, a subset of animals (*N*_animals_: 9EW‐Ctr: 6 + 5, SF‐Ctr 6 + 5) was euthanized by cervical dislocation, decapitated and trunk blood collected into EDTA‐coated tubes with protease inhibitors (Complete, Roche, Basel, Switzerland). The samples were centrifuged (10 min at 1300*g* in 4°C) and plasma samples stored at −80°C for subsequent quantification of corticosteroid concentrations with the Corticosterone AlphaLISA Detection Kit (Product number: AL3020 HV/C/F) from Perkin Elmer (Waltham, Massachusetts, USA).

### 2.6 Recording and analysis of EEG and EMG

To quantify the different vigilance stages and collect information on EEG oscillations, we implanted a total of six animals with telemetric transmitters (HDX02; Data Sciences International [DSI]; St. Paul, Minnesota, USA) as described previously in detail in (Steffens et al., 2022). Briefly, under general isoflurane (Attane Vet, Kansas City, Missouri) anesthesia, two biopotential leads were placed bilaterally in the epidural space for EEG in the frontal and parietal areas (0.2 mm lateral to midline, 0.8 mm anterior from Bregma; 1.5 mm lateral to midline, 3.4 mm posterior from Bregma, respectively), and two leads were placed in the nuchal musculature for EMG recordings. Ten days after the surgery, the recording with a sampling rate of 500 Hz (bandwidth: 0.5–80 Hz) was started. The recording system (DSI, New Brighton, Minnesota, USA) consisted of receiver plates (PhysioTel Receiver, model RPC‐1) placed beneath the cages, a DSI Matrix 2.0 (MX2). The data were collected with the DSI Talker interface to Spike2 (version 8.17; Cambridge Electronic Design (CED), Cambridge, UK). After a 10‐day recovery period, the baseline recordings (48 h) preceding the treatments were started.

The collected EEG/EMG data were then scored for vigilance stages with the help of our machine‐learning algorithm first published in (Rytkönen et al., 2011) and recently updated (12/23) and made available for Spike2 users (autoscore 1.86.s2s: https://ced.co.uk/downloads/scriptspkanal). To train the algorithm, a subset of a recording was segmented into 4‐s epochs, and manually classified as wake, NREM, REM, or noise based on standard criteria (Mäkelä et al., 2010). After the initial training step, the algorithm automatically processed the rest of the file.

The vigilance stage‐specific EEG power spectral was then calculated on the processed files within the Nyquist‐compliant frequency range of 0.5–80 Hz by fast Fourier transform (FFT = 1024; Hanning window; 0.5 Hz resolution) and collected into 3 h time bins for wake, NREM, and REM using Spike2 analysis tools integrated into autoscore. Before further analysis, all EEG power spectra were normalized to the total power of the recording day for each individual animal to control for the nonspecific effects of recording instrumentation.

From the power spectrum, we focused on the following frequency bands:

1. Sleep: NREM delta (0.5–4 Hz); associated with sleep intensity and NREM homeostasis (Franken et al., 2001)
2. Wake:
  - Fast waking theta (8–12 Hz); associated with exploratory behavior and active waking, also referred to as TDW by Vassalli et al. (2017)
  - Waking gamma (30–80 Hz); associated with EEG waking theta and high‐level information processing (Franken et al., 2001)

The power bands are displayed as cumulated power following Stenberg et al. (2003) (power multiplied with the number of epochs per 3 h bin).

To avoid a microglial reaction to the EEG electrode implantation, we measured microglial morphology and vigilance stages in separate animals.

### 2.7 Statistical analysis

We tested the datasets for normality and homogeneity of variance using the Kolmogorov–Smirnov test. The effects of vigilance stages on extended wakefulness, the following recovery sleep, extended wakefulness, chronic SF, and EEG oscillations on microglial morphology features (Territory, Volume, Ramification Index) were evaluated using generalized estimating equations (GEEs). This method accounts for partial within‐subject dependencies, which is crucial given that we analyzed microglia cells based on sampling timepoints and brain area, rather than by individual animals. Additionally, we employed a planned post hoc Bonferroni method to correct for multiple testing. Results are presented as mean ± standard error of the mean (SEM); with *p* values <0.05 considered statistically significant.

## 3 RESULTS

### 3.1 Microglial morphology dynamically follows the vigilance stage‐specific brain oscillations

In our previous study, we used male B6 mice to follow‐up microglial morphology across a 24 h light–dark cycle and observed a decrease in all measured microglial features during the light period and particularly at zeitgeber time point 3 (ZT03) associated with the maximal delta power during NREM sleep.

To further investigate the possible interaction between vigilance stage‐specific brain oscillations and microglial morphology, we compared microglial morphology during periods of higher frequency brain oscillations (theta and gamma) to periods of NREM sleep (delta) oscillations. We found that microglia exhibit larger volume and territory coverage, as well as more complex ramification patterns, during waking phases characterized by higher frequency activity (Figure 1b). This finding supports the hypothesis that waking theta and gamma activity may be associated with increased microglial complexity, in contrast to the reduced complexity observed during NREM sleep, which is dominated by delta activity.

### 3.2 Out of reach: Microglial morphology in brain areas not directly connected to vigilance stage‐specific oscillations

To further investigate the influence of vigilance stage‐specific brain oscillations on microglial morphology, we examined the hindbrain areas that lack functional connections to these oscillations (Barmack, 2003). These areas included the CC, DCN, and MVN. We selected two timepoints that in areas functionally connected to the vigilance stage‐specific oscillations (i.e., SC, HC, BF) exhibit significant differences in microglial morphology, namely the ZT6 (minimum) and ZT18 (maximum) (Figure 1b,c).

Interestingly, none of the cerebellar/hindbrain areas displayed similar changes in microglial morphology. In fact, the MVN exhibited a pattern opposite to the cortical areas: microglia in this region increased their territory and ramification complexity (GEE and post hoc Bonferroni; *p* = 0.036 and *p* = 0.0256) during the light period at ZT6 compared with ZT18. This contrasting pattern suggests that microglial morphology changes in the MVN might be driven by different factors than those in cortical regions, possibly involving intrinsic circadian mechanisms.

### 3.3 Microglial morphology and sleep SWA are linked

#### 3.3.1 Extended active waking (3EW)

To investigate the relationship between microglia morphology and the vigilance stage‐specific EEG oscillations, we extended waking into the habitual sleeping phase by keeping the animals awake for 3 h (ZT0–ZT3) by introducing novel objects into their cages (gentle handling method). This procedure initiates exploratory behavior, and consequently enhances waking EEG power in the fast theta/gamma frequency ranges as compared with undisturbed controls (Figure 3c).

When comparing microglial morphology of 3EW to 3EW‐Ctr mice, we observed a statistically significant increase of ramification complexity in the SC (GEE and post hoc Bonferroni; *p* = 4.3521E‐14) and HC (GEE and post hoc Bonferroni; *p* = 1.10E‐04), as well as an increase in territory in the SC (GEE and post hoc Bonferroni; *p* = 6.80E‐05) (Figure 2b).

#### 3.3.2 SWA‐enriched recovery sleep

To further elucidate whether vigilance stages, and the vigilance stage‐specific EEG oscillations, were responsible for the observed effects on microglial morphology, we performed extended wakefulness for 4 h (ZT0–ZT4) to initiate subsequent SWA‐enriched recovery sleep for 2 h (ZT4–ZT6). When the EWrec mice were compared with their time‐matched (ZT6) untreated controls, we found no differences except de‐ramification in hippocampal microglia (Figure 2c), indicating that the morphological changes initially caused by extended wakefulness were restored by sleep. These findings suggest a connection between microglial morphology, sleep, and sleep oscillations, since they appear to play a role in maintaining or restoring microglial ramification complexity and territory.

### 3.4 Microglial morphology in sleep manipulations with minimal capacity to induce sleep SWA

Given the observed influence of sleep SWA on microglial morphology when both the homeostatic and circadian processes favor sleep (i.e., in the beginning of the light period), we also investigated the impact of sleep manipulations that only minimally induce SWA‐enriched recovery sleep without inducing a physiological stress response (Figure 3).

#### 3.4.1 Nine hours of extended wakefulness (9EW)

We compared effects of 9 h of extended wakefulness (9EW) via the gentle handling method to the microglial morphology of their control animals (9EW‐Ctr) (Figure 3a) and found a decrease in microglial ramification across all three examined brain areas (GEE and post hoc Bonferroni; SC *p* = 1.98E‐05; HC *p* = 2.1E‐9; BF *p* = 0.039), a reduction in microglial territory within the hippocampus (GEE and post hoc Bonferroni; *p* = 0.011), and an increase in microglial volume in the BF (GEE and post hoc Bonferroni; *p* = 0.001).

In contrast to the 3EW, we did not observe the same effects on microglial morphology after 9EW. In fact, we observed an opposite effect in terms of ramification complexity. It should be noted that SWA already gradually increases during 9EW, especially within the last 3 h (Figure 3c), which provides a likely explanation for the observed differences between microglia after the 3EW and 9EW treatments.

#### 3.4.2 Two weeks of SF

Comparing microglia after SF with their respective controls (Figure 3b), we did not find any changes of microglial ramification. However, HC microglia increased their territory (GEE and post hoc Bonferroni; *p* = 0.009) and BF microglia increased their volume (GEE and post hoc Bonferroni; *p* = 2.22E‐04).

## 4 DISCUSSION

Our findings reveal that microglia exhibit distinct morphological features that dynamically follow the vigilance stage‐specific brain oscillations. During the early light phase, typically dominated by NREM SWA, microglia displayed reduced morphological complexity. In contrast, during the dark phase, enriched with theta‐ and gamma‐dominated wakefulness, microglia exhibited increased morphological complexity.

To study the connection of microglial morphology with sleep *per se*, separated from the diurnal component, we extended wakefulness into the light period by 3 h. The intervention effectively shifted the animals' sleep‐onset time, and during the extension, replaced the SWA with wakefulness‐associated higher frequency oscillations. This manipulation resulted in partial recovery of the previously observed decline in microglial morphology features during sleep, suggesting that, in addition to diurnal changes (Steffens et al., 2022), the morphological changes are related to changes in brain oscillations. Previous studies by others support this view. Microglial interactions with synapses are activity‐dependent (Wake et al., 2009), and sensory deprivation or alteration lead to alterations in microglial behavior. For instance, Sipe et al. (2016) observed increased microglial process motility in the visual cortex following visual deprivation. Similarly, Tremblay et al. (2010) reported that deprivation and reexposure induced a range of microglial responses, including morphological changes, altered extracellular space, phagocytosis, and increased synaptic interactions. Finally, Liu et al. (2019) demonstrated that reductions in local neuronal activity through sensory deprivation or optogenetic inhibition resulted in an enlarged microglial surveillance territory. Aligning with our findings, this suggests that microglial morphology changes might reflect their dynamic responses to neuronal activity fluctuations throughout the sleep–wake cycle.

Hristovska et al. (2022) explored the relationship between neuronal activity and microglial morphology during sleep. Their study employed mice lacking Cx3CR1, a microglial receptor crucial for neuron–microglia communication (Sheridan & Murphy, 2013). Notably, these mice did not exhibit the typical reduction of microglial complexity and motility observed during NREM sleep compared with wakefulness. This finding suggests that the link between neuronal activity and microglial dynamics may be mediated by CX3CR1 signaling. Our findings of reduced microglial complexity during sleep further support this potential link. A recent study by Gu et al. (2023) used *in vivo* imaging in the SC during recorded sleep–wake periods and also found evidence of morphological changes in microglia, however, the changes were contrary to what we and the previously cited studies found. Whether this is due to differences in methodology (the up to 1 h‐long imaging periods conducted *in vivo* might have triggered microglia to adopt their immuno‐reactive, amoeboid shape as reported) or failure to separate the circadian and homeostatic components in the data collection, or other important methodological aspects, remains to be clarified in future experiments.

The wake‐specific neuronal activity is associated with elevated glutamatergic activity. It has been previously shown that ionotropic glutamatergic signaling increases microglial ramification (Fontainhas et al., 2011). Glutamatergic neuronal activity increases ATP metabolism and increases extracellular adenosine (Wigren et al., 2007). Adenosine, an inhibitory neuromodulator, directly inhibits neuronal activity, and in sleep regulatory areas contributes to sleep promotion (Porkka‐Heiskanen et al., 1997). Thus, one of the mechanisms postulated to induce the homeostatic sleep response, or “sleep pressure”, is purinergic signaling. Generally, ATP leads to a significant increase in microglial morphological complexity (Fontainhas et al., 2011; Pocock & Kettenmann, 2007), resembling wake‐related morphology. Another link between microglia and sleep is the microglial expression of the ectoenzymes CD39 and CD73, as well as nucleoside transporters. These enzymes and transporters facilitate catabolism of ATP and ADP to adenosine (Matyash et al., 2017) and thus contribute to sleep promotion.

A potential mediator for the interaction between sleep‐specific neuronal activity and microglial morphology may be the neurotransmitter γ‐aminobutyric acid (GABA) (Logiacco et al., 2021). Previous *in vitro* studies have shown that an increase in GABAergic signaling tends to decrease microglial process length and motility (Fontainhas et al., 2011), resembling changes observed during sleep. In the presence of extracellular ATP, GABA can cause microglial processes to elongate (Fontainhas et al., 2011). This suggests that an interplay of GABAergic, glutamatergic and purinergic signaling is a potential driver for the morphological changes observed in microglia, where molecules with increased concentrations during waking (glutamate, ATP) are associated with complex microglial morphology, while sleep‐related molecules (GABA) are associated with decreased complexity. Additionally, there could be a reciprocal interaction between microglia and neuronal activity since neuronal GABA induces microglia to release cytokines (such as TNF‐α, IL‐6, and IL‐1β) (Lang et al., 2020), which also possess sleep‐inducing properties (Rockstrom et al., 2018).

Focusing on awake versus anesthetized animals, Stowell et al. (2019) demonstrated that noradrenergic levels modulate microglial function and morphology via microglial β2‐adrenergic receptors. While using anesthesia can also provide valuable experimental information on brain processes taking place in sleep, it is evident that anesthesia does not equal natural sleep, and particularly the intricate interplay between different vigilance stages and diurnal factors is lost in those experiments (Franks & Wisden, 2021).

In a series of experiments, we compared the effects of different sleep restriction protocols on microglial morphology. We selected protocols, which, while all shortened or disturbed sleep, were known to induce different homeostatic responses (Franken & Dijk, 2023; Ramesh et al., 2012).

Extended wakefulness for 3 h increased microglial morphological complexity in both the SC and hippocampus, as compared with controls. The changes resemble the morphology features of microglia induced by wake‐specific neuronal activity, as described above. However, the 9 h continuous extension of wakefulness induced a decrease in microglial ramification complexity within the SC, HC, and BF, resembling somewhat sleep‐related, low neuronal activity morphology. Bellesi et al. (2017) reported no changes in microglial ramification complexity or density in the frontal cortex after 8 h of sleep deprivation/extended wakefulness. These discrepancies may be attributed to differences in the investigated brain regions. Importantly, neither of the studies evidenced an increase in wake‐type morphological features in microglia, despite the animals being awake continuously for 9 h.

The chronic SF (14 days) protocol offers a more translationally relevant model, mimicking human sleep disorders like sleep apnea and insomnia. This protocol induced only minor changes in microglial territory (HC) and volume (BF), in accordance with previously reported results (Bellesi et al., 2017; Hall et al., 2020). This protocol does not reduce the overall time spent in NREM sleep, it only disrupts the sleep episodes (Ramesh et al., 2009).

What could explain the differences in microglial response to the different sleep restriction protocols? It has been suggested that different protocols induce different levels of stress. In the present study, neither of the sleep manipulation protocols induced significant elevations in corticosterone levels (Figure 3d), suggesting the absence of acute physiological stress, as defined by elevated corticosterone levels.

One significant difference between the protocols is that while the 3EW protocol induces a robust homeostatic response, as evidenced by increased SWA during recovery sleep, this is not the case with the 9EW (Franken & Dijk, 2023) or SF protocol (Trammell et al., 2014). It can be speculated that while the short wake extension protocol is able to substantially increase cortical neuronal activity (intensity of waking) (Huber et al., 2007), during longer waking periods, maintenance of such heightened activity is no longer possible, which dilutes the homeostatic drive and induction of recovery SWA. The factors responsible for mediating the effect of increased neuronal activity to induce recovery sleep could be the same that drive the observed decrease in microglial ramification complexity. One potential candidate for such a factor is adenosine, a well‐established sleep‐promoting molecule (Porkka‐Heiskanen et al., 1997), as already discussed above. Levels of adenosine rise during wakefulness, rising further during prolonged wakefulness and subsequently decrease during sleep (Basheer et al., 2000; Dworak et al., 2010; Porkka‐Heiskanen et al., 1997). Another important candidate for such mediators is cytokines. Prolonged wakefulness triggers the release of various cytokines, some of which are sleep‐promoting (Rockstrom et al., 2018) and are also sensed by microglia (Hanisch, 2002). Since neither adenosine nor cytokine levels were measured in this study, these questions remain open and can only pave the way for further studies.

Interestingly, a recent study by Mattei et al. (2024) compared the microglial transcriptome and proteome between the light and dark phases. They found increased expression of P2Y12 and CD39 during the wake phase (ZT18 vs. ZT6), along with genes and proteins associated with cytoskeletal dynamics. This finding provides a molecular basis for the morphological changes we observed in our work in relation to neuronal activity.

The role of circadian rhythms in regulating microglia morphology has been previously studied. Most studies were carried out under light/dark conditions (Bellesi et al., 2017; Hristovska et al., 2022), including our previous study (Steffens et al., 2022), consequently failing to separate between circadian and vigilance stage‐driven morphology changes. A few truly circadian studies conducted under constant darkness have been published (Griffin et al., 2019; Hayashi, Koyanagi, Kusunose, Okada, et al., 2013; Hayashi, Koyanagi, Kusunose, Takayama, et al., 2013). The daily variations found by Hayashi, Koyanagi, Kusunose, Takayama, et al. (2013) were absent in mice deficient in the core circadian clock gene, *Clock*, and in animals administered with a purinergic P2Y12 receptor antagonist, indicating that the circadian regulation of microglial morphology is dependent on both the functional *Clock* gene and intact purinergic signaling.

To investigate differences in regional changes in microglial morphology and their potential connection with vigilance stage‐dependent thalamocortical oscillations, we also examined microglia in the hindbrain (MVN, DCN) and cerebellum (CC), regions lacking direct functional connections to the areas responsible for the generation of vigilance stage‐dependent thalamocortical oscillations. In these regions, we did not observe the same diurnal and vigilance stage‐dependent patterns of microglial morphology as those observed in areas inherently involved in the generation of vigilance stage‐specific brain oscillations (SC) or with direct functional connections to them (HC and BF). This finding by no means excludes the possibility that microglia may follow their own intrinsic circadian rhythms (Fonken et al., 2015; Hayashi, Koyanagi, Kusunose, Okada, et al., 2013; Hayashi, Koyanagi, Kusunose, Takayama, et al., 2013; Nakanishi et al., 2021) that in some areas is synchronized with vigilance stage‐dependent oscillations, and in other areas is not. Differences in microglial morphology between different areas have been previously reported (Ni et al., 2024). In this connection, it may be noted that the EEG signal is primarily limited to measuring thalamocortical oscillations, and thus other possible brain oscillations remain outside the scope of this study.

One key question is how these intrinsic rhythms synchronize within the brain's vigilance stage‐dependent oscillations and with the larger circadian network. A potential candidate for this synchronization could be melatonin, a hormone secreted by the pineal gland. Melatonin's primary physiological function is to convey information regarding the light–dark cycle to various bodily processes, including brain physiology (Cajochen et al., 2003). Microglia express MT1 and MT2 receptors specific for melatonin, which upon activation suppress proinflammatory signals and promote anti‐inflammatory ones (Gao et al., 2020; Gu et al., 2021). Furthermore, melatonin likely influences microglia through interaction with astrocytes and neurons, influencing the overall inflammatory response (Hardeland, 2021). However, we currently lack concrete knowledge about melatonin's involvement in the synchronization of microglial circadian rhythms.

The regional differences in microglial morphology originate from many potential factors. Microglia adapt their function to the local environment, leading to regional heterogeneity (Lawson et al., 1992; Tan et al., 2020; Verdonk et al., 2016). This adaptation is likely influenced by factors such as the presence of specific neuronal populations, the density of synapses, and the unique metabolic demands of different brain regions (Kierdorf & Prinz, 2017; Tan et al., 2020). For instance, cerebellar microglia exhibit a heightened capacity for engulfment and catabolism of cellular debris, whereas microglia in the striatum display a focus on homeostatic surveillance (Ayata et al., 2018). So far, little is known about microglia in the cerebellum (larger by their cytoplasmic volume and reduced ramification) and hindbrain (low levels of fractalkine expression) (Tan et al., 2020).

The most important finding of our study was that microglial morphology seems to follow the sleep SWA (an EEG marker of sleep homeostasis) in brain areas generating (or functionally connected to) the vigilance stage‐specific brain oscillations: microglial morphological complexity is minimal when SWA is maximal, and when we experimentally shifted the timing of maximal SWA, the connection of microglial morphology to SWA remained. Since we, and others (Hayashi, Koyanagi, Kusunose, Okada, et al., 2013; Hristovska et al., 2022; Nakanishi et al., 2021), have previously demonstrated that microglia display diurnal/circadian variation in morphology, it is intriguing to speculate that both elements of the 2‐process model of sleep regulation are also reflected in microglia morphology/function. Further studies concentrating on the interplay of circadian and neural modulation of microglia are warranted.

While this study sheds new light on the effect of sleep and wakefulness on microglia, it is important to acknowledge some limitations. The study, as most studies on relationships between microglia and sleep, was conducted solely on male mice. Interesting differences between the sexes may exist, as exemplified by the study where microglia depletion in female mice increased NREM sleep duration and number of episodes (Picard et al., 2023), while in males microglial depletion induced by PLX5622, a colony‐stimulating factor 1 receptor (CSF1R) inhibitor, induced an increase of overall sleeping time during the light period in the first week following complete microglial depletion (Rowe et al., 2022). Further, while our study showed a correlation between microglial morphology and thalamocortical oscillations, the underlying molecular mechanisms remain unexplored.

It can be noted that some of the key questions of the basic physiology of brain microglia remain unknown. These include the extent and role of regional specificity and its potential functional consequences. Further, while the role of microglia as part of the brain defense mechanism has been established, its role in neural communication remains to be firmly established and the mechanisms to be identified.

The question of whether a specific microglial morphological change is associated with a certain function, and/or whether microglia with a certain shape are limited to a particular role, remains unanswered. Furthermore, an inconsistent classification across research, despite studies linking microglial morphology to function (i.e., amoeboid microglia are associated with phagocytosis of cellular debris) (Graeber, 2010; Helmut et al., 2011; Li & Barres, 2018), makes comparisons challenging (Reddaway et al., 2023).

Our study employed static microglial imaging, which provided valuable data on the correlation between microglial morphology and sleep stages. However, future investigations could benefit from incorporating more dynamic approaches. Time‐lapse imaging would allow us to capture microglial behavior over time, offering a deeper understanding of their activity patterns during sleep and wakefulness. Additionally, employing higher resolution techniques like multi‐photon *in vivo* imaging could reveal finer details of microglial morphology. Finally, incorporating 3D reconstruction methods like 3D‐serial electron microscopy would provide a more comprehensive picture of microglial structure. These advancements would enable us to explore the dynamic changes in microglial morphology with greater precision.

Overall, our findings on microglial morphology changes between wake and sleep stages provide valuable insights into the dynamic nature of these cells and their potential role in sleep regulation. In the context of disease models, investigating microglial changes between wake and sleep is crucial since these cells are increasingly recognized as key players in both sleep homeostasis and neurodegenerative processes. Understanding their dynamic responses during these states could provide insights into how sleep disruption and abnormal microglial function contribute to disease progression. Further research is warranted to explore the potential therapeutic targets that might modulate microglial activity to promote healthy sleep patterns and potentially slow down neurodegeneration.

## AUTHOR CONTRIBUTIONS

*Study conceptualization, design and revision of the manuscript*. Henna‐Kaisa Wigren and Tarja Stenberg. *Project administration, data curation, analysis, visualization, statistical analysis, manuscript draft*. Sarah Steffens. *Hindbrain and cerebellar data curation*: Hilla Mäkinen. *Funding acquisition*: Tarja Stenberg, Sarah Steffens and Henna‐Kaisa Wigren.

## FUNDING INFORMATION

Finnish Society of Science and Letters, Signe och Ane Gyllenbergs stiftelse, Finska Läkaresällskapet I Finnish Medical Association, SLEEPWELL I Research Program I Faculty of Medicine, University of Helsinki, Doctoral Programme of Brain & Mind, University of Helsinki.

## CONFLICT OF INTEREST STATEMENT

The authors declare no conflicts of interest.

## Supporting information


**Table S1.** Overview of the numbers of all animals and cells analyzed per treatment and brain area.

## Data Availability

The data that support the findings of this study are available from the corresponding author upon reasonable request.
