# Supplementary figures and images for: Microglial morphology aligns with vigilance stage‐specific neuronal oscillations in a brain region‐dependent manner
Source: Glia. 2024 Sep 20;72(12):2344–56. doi: 10.1002/glia.24617 (PMC13058872; doi:10.1002/glia.24617)

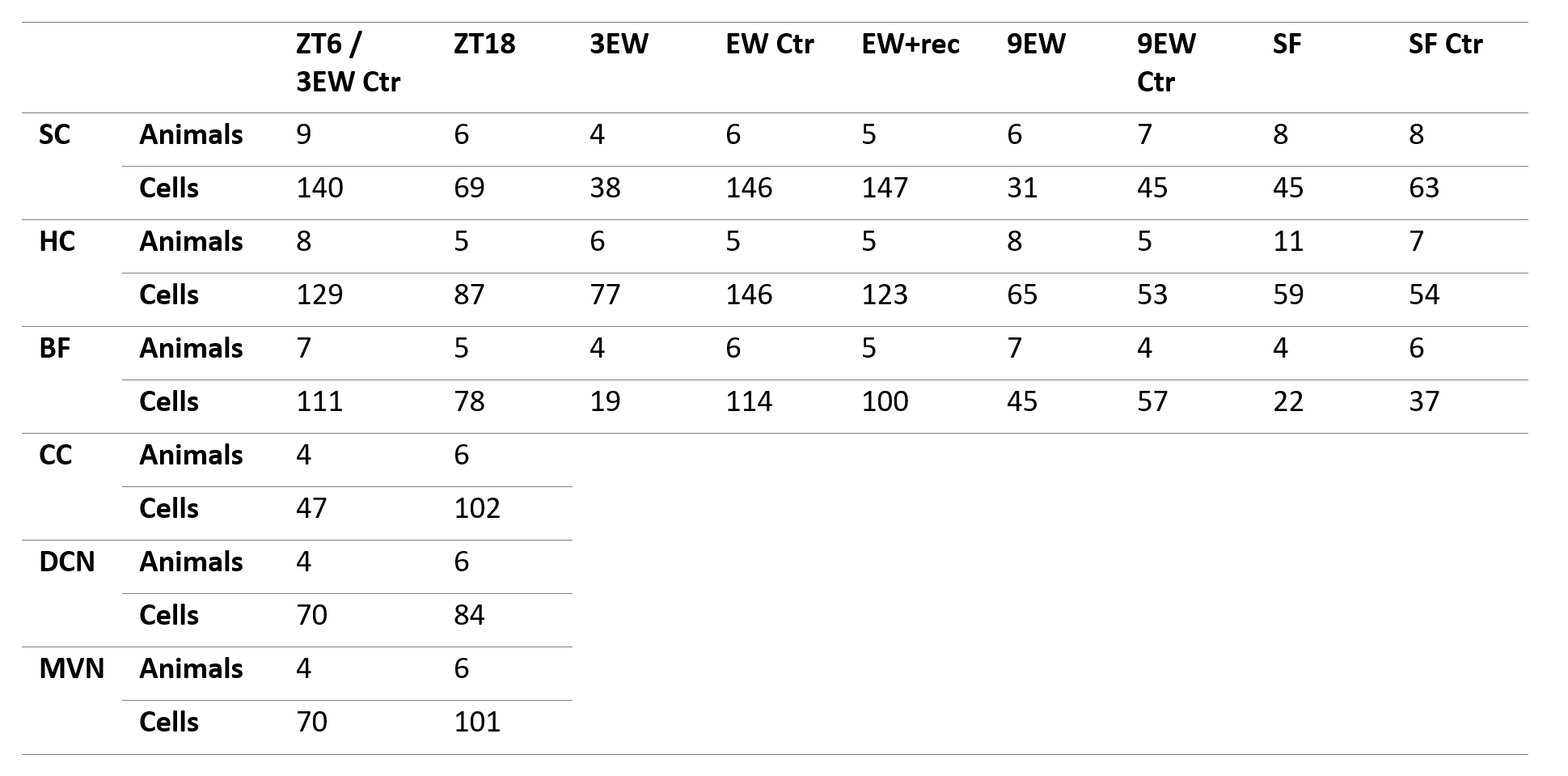

Supplement: Supplementary file 1 — Table S1. Overview of the numbers of all animals and cells analyzed per treatment and brain area. [file GLIA-72-2344-s001.PNG]
